# Prognostic and diagnostic value of epithelial to mesenchymal transition markers in pulmonary neuroendocrine tumors

**DOI:** 10.1186/1471-2407-14-855

**Published:** 2014-11-20

**Authors:** Jose A Galván, Aurora Astudillo, Aitana Vallina, Guillermo Crespo, Maria Victoria Folgueras, Maria Victoria González

**Affiliations:** Tumor Bank Laboratory, University Institute of Oncology Principality of Asturias, CajAstur Welfare Project (IUOPA, c/ Celestino Villamil s/n, 33006 Oviedo, Asturias, Spain; Pathology Department, University Central Hospital of Asturias, 33006, Oviedo, Spain; Oncology Department, University Hospital of Burgos, c/ Islas Baleares, 3, 09006 Burgos, Spain; Surgery Department, Faculty of Medicine and Health Sciences, University of Oviedo, c/ Julián Clavería s/n, 33006 Oviedo, Asturias, Spain; CajAstur Welfare Project (IUOPA), University Institute of Oncology Principality of Asturias, c/ Julián Clavería s/n, 33006 Oviedo, Asturias, Spain

**Keywords:** Pulmonary neuroendocrine tumors, Epithelial-Mesenchymal transition, E-cadherin, β-catenin, Snail, Foxc2

## Abstract

**Background:**

Pulmonary neuroendocrine tumors (Pulmonary NETs) include a wide spectrum of tumors, from the low-grade typical carcinoid (TC) and the intermediate-grade atypical carcinoid (AC), to the high-grade large-cell neuroendocrine carcinoma (LCNEC) and the small-cell carcinoma (SCLC). Epithelial Mesenchymal Transition (EMT) is a process initially recognised during several critical stages of embryonic development, which has more recently been implicated in promoting carcinoma invasion and metastasis. The initial stage of the EMT process begins with the deregulation of adhesion molecules, such as E-cadherin, due to transcriptional repression carried out by factors such as Snail family members, Twist and Foxc2.

**Methods:**

Immunohistochemistry for EMT markers and E-cadherin/ β-catenin complex in 134 patients with pulmonary NETs between 1990 – 2009. Analysis of potential associations with clinicopathological variables and survival.

**Results:**

Pulmonary NETs of high malignant potential (LCNEC and SCLC) had reduced expression of the adhesion molecules and high level expression of transcriptional repressors (Snail1, Snail2, Twist and Foxc2). Snail high expression levels and the loss of E-cadherin/β-catenin complex integrity had the strongest negative effect on the five-year survival rates. E-cadherin/β-catenin complex integrity loss independently predicted lymph node involvement and helped in Atypical Carcinoid (AC) vs Typical Carcinoid (TC) differential diagnosis. Importantly, among the TC group, the loss of E-cadherin/β-catenin complex integrity identified patients with an adverse clinical course despite favourable clinicopathological features.

**Conclusion:**

The immunohistochemical determination of E-cadherin/β-catenin complex integrity loss and EMT markers in the clinical setting might be a potential useful diagnostic and prognostic tool especially among the TC patients.

**Electronic supplementary material:**

The online version of this article (doi:10.1186/1471-2407-14-855) contains supplementary material, which is available to authorized users.

## Background

Pulmonary Neuroendocrine Tumors (Pulmonary NETs) express differential characteristics of neuroendocrine cells scattered among epithelial cells [1, 2]. They represent a broad clinico-pathologic spectrum and have variable morphologic features and biologic behaviours. Pulmonary NETs account for 20% of lung carcinomas and their incidence has increased significantly in recent decades (6% per year), due in part to early diagnosis imaging; however, most of them are found accidentally [3–5].

The current classification of the World Health Organization (WHO), 2004, for pulmonary NETs, proposed by Travis W. D. et al. [6], defines four histological types based on conventional morphological data (organoid growth pattern, mitotic index and necrosis) and immunohistochemical features, with different prognostic and therapeutic implications: 1. Typical Carcinoid (TC): “well differentiated neuroendocrine tumors of low malignant potential”. 2. Atypical Carcinoid (AC), “well-differentiated neuroendocrine tumors of intermediate malignant potential, 3. and 4. Large Cell Neuroendocrine Carcinoma (LCNEC) and Small Cell Lung Carcinoma (SCLC), both poorly differentiated neuroendocrine tumors of high malignant potential.

Epithelial-to-Mesenchymal Transition (EMT) is a reversible process of cellular changes including loss of apico-basal polarity [7], disintegration of tight junctions [8] and acquisition of a variable cell shape that facilitates cell movement and metastasis [9, 10]. Reduction of cell–cell adherence is achieved via the transcriptional repression and delocalization of cadherins [11, 12]. Members of the Snail family (Snail1 and Snail2), induce EMT by repressing the transcription of E-cadherin [13, 14], similarly to the mechanism of action of Twist [15, 16], and indirectly Foxc2 [17]. Moreover, when E-cadherin is deregulated, β-catenin is detached from the cell membrane and translocated to the nucleus to participate in EMT signalling events [18]. During EMT, another process called “cadherins switch” takes place. It has been described as an increase in N-cadherin expression (neural cadherin), with or without decrease of E-cadherin [19, 20].

In 2010, our group showed evidence that Snail expression is associated with pulmonary NETs malignancy potential [21]. In the present study, we analyze other EMT markers in a larger cohort of patients with pulmonary NETs, their relation with E-cadherin/β-catenin complex expression and their associations with molecular and relevant clinicopathologic features.

## Methods

### Patients and samples

134 surgical pulmonary NETs samples (diagnosed between 1990 and 2009) were obtained from the Pathology Unit of the Hospital Universitario Central de Asturias, the Instituto Nacional de Silicosis and the Centro Médico de Asturias. Informed consent approved by the Hospital Ethical Board (Comité ético de investigación clínica regional del Principado de Asturias) for sample banking and research use was obtained from patients at the time of the surgery. The series included 66 TCs, 10 ACs, 18 LCNECs and 40 SCLCs transbronchial biopsies. Clinicopathologic features for the entire cohort of patients are summarized in Table 1.

**Table 1:** **Clinicopathologic features of the studied patients and their tumors (N = 134)**

	N	%
**Age (Mean 56 years)**		
≤ 56 years	61	45.5
> 56 years	73	54.5
**Gender**		
Female	45	33.6
Male	89	66.4
**Lymph nodes status**		
Free	84	62.7
Affected	45	33.6
Unknow	5	3.7
**Tumor size (Mean 3 cm)**		
Small ≤3 cm	80	59.7
Big >3 cm	54	40.3
**Necrosis**		
Negative	68	50.7
Positive	44	32.8
Unknow	22	16.4
**Mitotic index**		
≤2 mitosis	64	47.8
3-20 mitosis	11	8.2
>20 mitosis	35	26.1
Unknow	24	17.9
**Diagnosis WHO**		
TC	66	49.3
AC	10	7.5
LCNEC	18	13.4
SCLC	40	29.9
**Tobacco consumption**		
Non-smorker	52	38.8
Smorker	82	61.2
**Treatment**		
Surgery	98	73.1
Chemotherapy	57	42.5
Radiotherapy	31	23.1

The diagnosis of neuroendocrine tumors was based on morphologic criteria according to the most recent World Health Organization classification and on immunophenotypical findings, including reactivity to neuroendocrine markers (synaptophysin and chromogranin A) as well as the evaluation of proliferation marker Ki67 (Additional file 1).

### Tissue microarrays construction

Tissues obtained from surgical specimens were fixed in 10% formaldehyde and paraffin embedded, and stained with H&E. Representative tumor regions were selected to make four tissue microarrays (TMA) containing three tissue cores from each of the 134 samples. Microarrayer (Beecher Instruments, Sun Prairie, WI, USA) was used. After five minutes at 60°C, the TMA blocks were cut in 4 μm-thick sections for immunohistochemical techniques.

### Immnunohistochemistry

The automated system DISCOVERY® (Ventana Medical System, Tuczon, AZ, USA) was used to carry out the immunohistochemical protein detection of interest. Deparaffinized sections were rehydrated in EZ Prep® (Ventana Medical System, Tuczon, AZ, USA) for 20 minutes. Antigen retrieval was done by heating citrate buffer solution (pH 6.5) and HCl-Tris buffer solution (pH 9.0). Non-specific antibody binding was blocked using casein (Antibody block®, Ventana Medical System, Tuczon, AZ, USA) for 20 minutes. Endogenous peroxidase activity was blocked with H_2_O_2_ solution (Inhibitor®, Ventana Medical System, Tuczon, AZ, USA) for four minutes. Samples were incubated with primary antibody at 37°C: polyclonal anti-Snail1 (Abcam 17732, Cambridge, UK) (1:300 dilution); polyclonal anti-Snail2 (Abcam Cambridge, UK) (1:100 dilution); polyclonal anti-Twist (Abcam Cambridge, UK) (1:1000 dilution); polyclonal anti-Foxc2 (ABR Affinity Bioreagents, Colorado, USA) (1:600 dilution), monoclonal anti-E-cadherin (Dako, Denmark) (1:100 dilution) and monoclonal anti-β-catenin (Sigma, Missouri, USA) (1:1000 dilution), monoclonal anti-N-cadherin (Becton Dickinson, NJ, USA) (1:200 dilution) and monoclonal anti-Vimentin (Ventana Medical System, Tuczon, AZ, USA) (1:100 dilution).

The slides were incubated with the secondary antibody (OmniMap® Ventana medical System, Tuczon, AZ, USA) for 30 minutes at room temperature. Subsequently, the samples were visualized with DAB (3-3’-Diaminobenzidine) (Ventana Medical System, Tuczon, AZ, USA). Finally, samples were counterstained with hematoxylin (Ventana Medical System, Tuczon, AZ, USA), dehydrated and mounted in Entellan® (Merck, Germany). The sections were studied and photographed under a light microscope (20× objective, Nikon - Eclipse 80i, Japan).

The healthy respiratory epithelium was taken as positive control for E-cadherin and β-catenin and as negative control for all transcription factors implicated in EMT.

The protein expression levels were evaluated by two independent observers, AA and JAG. The interobservers and intraobserver reproducibility of IHC staining scoring was determined by Kappa statistics for all markers assessed by two independent observers (Additional file 2). In case of discrepancy a consensus was reached with the help of a third observer (MVG). Two parameters were taken into account: immunohistochemical signal intensity (in a 0-3 point scale) and the percentage of positive tumor cells in a 20× field (1.2 mm). The product of both parameters rendered a score for each specimen. For statistical purposes, the tumors were divided into two groups, taking the median score value for each marker as a cut-off point.

For both cadherins and β-catenin, their presence/absence in the cell membrane was recorded. We created a variable reflecting the integrity of the E-cadherin/β-catenin complex in the cellular membrane, which included two categories: retained integrity (both molecules showing a membranous pattern) and lost integrity (expression of at least one of them not observed in the membrane). For EMT markers (Snail1, Snail2, Twist and Foxc2) only nuclear immunostaining signal was considered and Vimentin expression was assessed as present *vs*. absent staining.

### Statistical analysis

The experimental results distributed among the different clinical groups of tumors were tested for significance employing the χ^2^ test (with Yates’ correction, when appropriate) or logistic regression models to evaluate the independent effect (Odds Ratio) of transcription factors on a given clinicopathological or molecular feature.

Survival curves were calculated using the Kaplan-Meier product limit estimate. Differences between survival times were analyzed by the log-rank method and the Hazard Ratio was calculated by univariate Cox regression analysis.

Multivariate Cox proportional hazard models (forward Wald method) were used to examine the relative impact of those statistically significant variables in univariate analysis. All statistical analysis was carried out with the software package SPSS 20.0 (SPSS, Inc., Chicago, IL). All tests were two-sided and p < 0.05 values were considered statistically significant.

## Results

The results are reported following the REMARK guidelines (REporting recommendations for tumor MARKer prognostic studies) [22].

### Associations between molecular findings and clinicopathologic features

**Figure 1:**
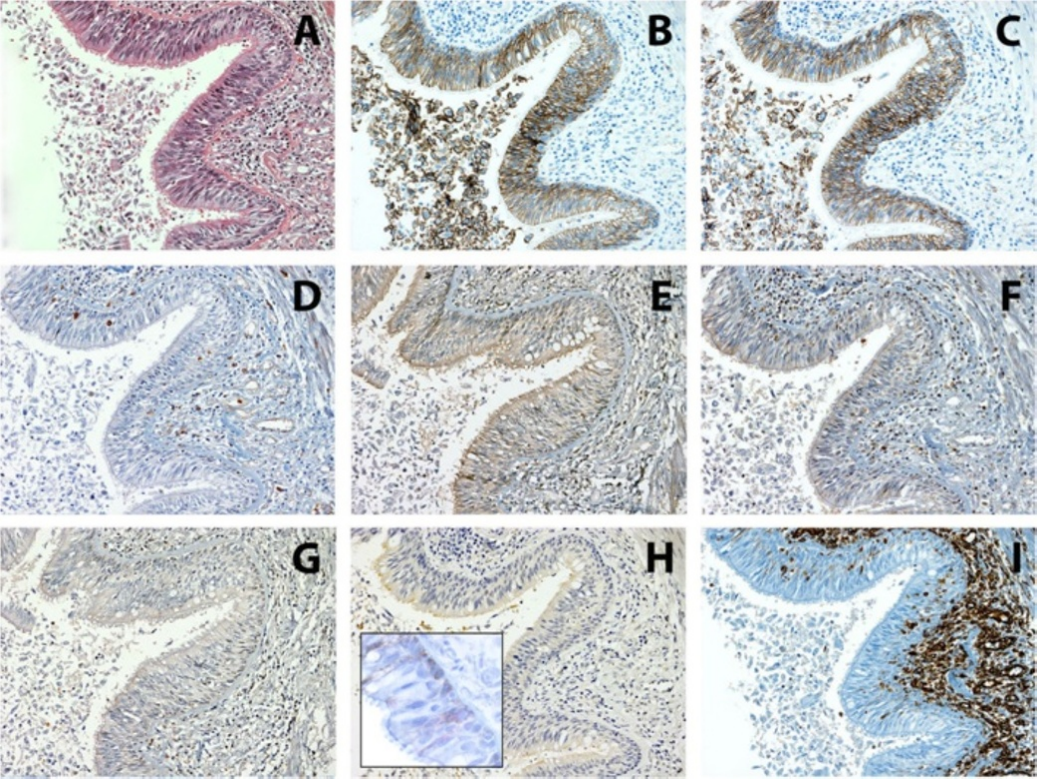
**Immunohistochemistry of EMT markers in healthy respiratory epithelium. A**: H&E, **B**: E-cadherin, **C**: β-catenin, **D**: Snail1, **E**: Snai2, **F**: Twist, **G**: Foxc2, **H**: N-cadherin (inset image, shows weak positive N-cadherin signal) and **I**: Vimentin. 50 μm scale bar (200X).

**Figure 2:**
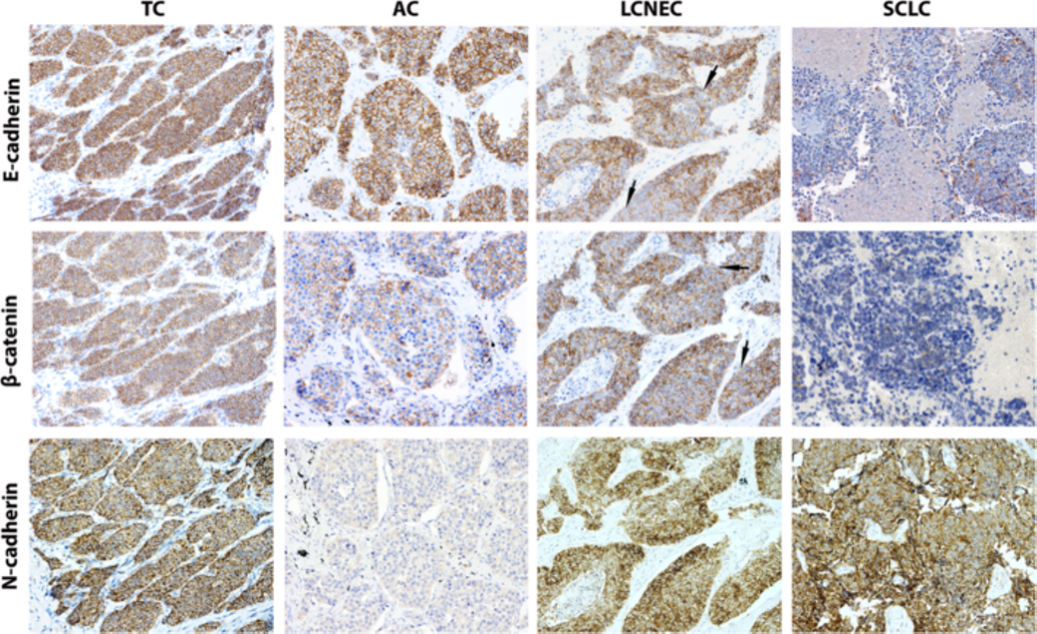
**Immunohistochemistry of E-cadherin, β -catenin and N-cadherin protein expression (rows) in pulmonary NETs (columns) 50 μm scale bar (200X).** Consecutive tissue sections of each NET type are shown for each marker. Arrows, disrupted membranous pattern for Ecadherin/β-catenin signal.

Expression of adhesion molecules (E-cadherin, β-catenin), was detected in the healthy epithelial tissue (Figure 1B, 1C) with a faint positive N-cadherin signal (Figure 1H, inset). In tumours, reduced expression of E-cadherin and β-catenin was observed and this was associated with high malignant potential tumours (p = 0.0001 for all). Moreover, E-cadherin and β-catenin were localized more frequently in the cytoplasm, so that E-cadherin/β-catenin complex loss of integrity was associated with LCNECs and SCLCs (p = 0.0001). Regarding N-cadherin, the signal intensity decreased with increasing tumor malignancy (Figure 2).

In addition, the integrity of E-cadherin/β-catenin complex was lost in the unfavourable categories of these clinicopathologic variables: tumor size (>3 cm) (p = 0.006), presence of lymph node metastasis (p = 0.0001), presence of necrosis (p = 0.0001), higher mitotic index (p = 0.0001) and tobacco consumption (p = 0.0001).

**Figure 3:**
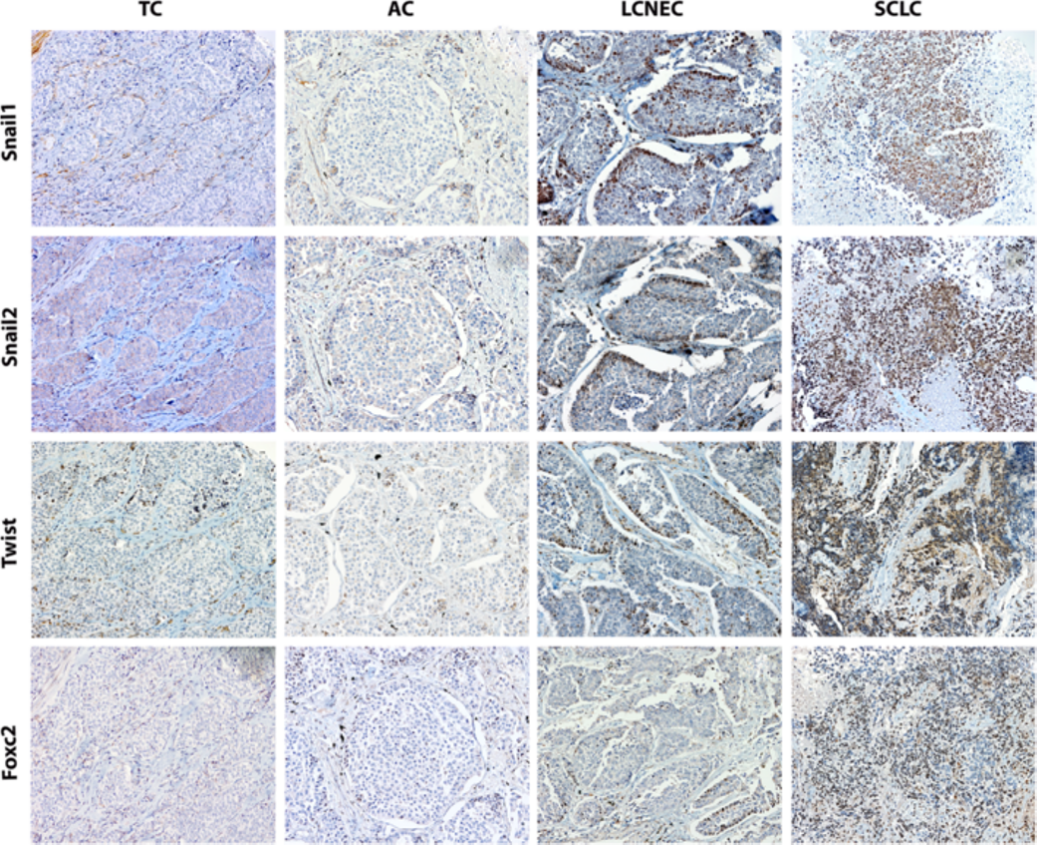
**Immunohistochemistry of Snail1, Snail2, Twist and Foxc2 protein expression (rows) in pulmonary NETs (columns) 50 μm scale bar (200X).**

In 39 cases β-catenin was not found at the membrane. 8 of these cases displayed a nuclear localization, corresponding to 6 SCLC, 1 LCNEC and 1 AC (Additional file 3).None of the transcriptional repressors (Snail1, Snail2, Twist and Foxc2) were expressed in the healthy respiratory epithelium (Figure 1D-1G). In tumors, all of them showed higher protein expression levels in high grade tumors (p = 0.0001 each) (Figure 3), with necrosis (p <0.05 each), a high mitotic index (p <0.001 each), in patients with lymph node involvement (p <0.01 each), and in smokers (p <0.01 each). In addition, larger tumors had higher Snail1 expression (p = 0.009). The mesenchymal marker Vimentin was expressed at higher levels in high grade tumors (p = 0.012).

Among Snail1, Snail2, Twist, Foxc2 and E-cadherin/β-catenin complex integrity, the following factors were independent predictors of lymph node involvement: SNAIL2 (OR: 4.9; 95% CI: 1.70–14.60, p = 0.003), TWIST (OR: 3.10; 95% CI: 1.11–8,64, p = 0.031), and E-cadherin/β-catenin complex integrity (OR: 5.64; 95% CI: 1.78–17.87, p = 0.003).

Mitotic index and tobacco consumption independently predicted lymph node involvement when they were included along with tumor size and necrosis in a logistic regression model (OR: 11.08; 95% CI: 1.08-113.95, p = 0.043 and OR: 6.36; 95% CI: 1.12-36.06, p = 0.037, respectively).

### Differential diagnosis between Pulmonary NETs subtypes

With the aim of identifying molecular variables useful in tumor subtypes discrimination, comparisons of each variable were made between TC *vs.* AC and LCNECs *vs.* SCLCs. We found significant differences in the patterns of different adhesion molecules, which were more frequent in TCs *vs.* ACs: membrane pattern of E-cadherin (p = 0.0001), of β-catenin (p = 0.032), of N-cadherin (p = 0.011) and E-cadherin/β-catenin complex integrity retained (p = 0.009). Figure 2 illustrates these findings regarding ACs vs TCs. With respect to N-cadherin, half ACs cases expressed low levels, as the one shown in the figure. Consecutive tissue sections for each marker were included.

Among the variables that differentiated SCLCs from LCNECs, high Snail1 (p = 0.0001), high Snail2 (p = 0.0001) and high Twist (p = 0.001), as well as reduced E-cadherin (p = 0.001), β-catenin cytoplasmic expression (p = 0.0001) and cytoplasmic pattern of N-cadherin (p = 0.001), were more frequent in SCLC *vs.* LCNEC.

### Molecular findings correlations

As expected, we observed an inverse correlation between E-cadherin and Snail1 (p = 0.02) or Snail2 protein expression levels (p = 0.001). In addition, the E-cadherin localization in the membrane correlated when EMT markers low expression: Snail1 (p = 0.0001), Snail2 (p = 0.0001), Twist (p = 0.013) and Foxc2 (p = 0.002).

In a logistic regression model including Snail1, Snail2, Twist and Foxc2, high Snail1 expression levels conferred an elevated risk of losing complex integrity (OR 4.91; 95% CI: 1.909 – 13.348, p = 0.002), and an elevated risk of finding N-cadherin in the cytoplasm (OR 5.9; 95% CI: 2.002 – 17.916, p = 0.001). A similar model rendered Twist as the transcription factor whose expression correlated with N-cadherin expression (OR 2.8, 95% CI: 1.24 – 6.414, p = 0.016).

Vimentin expression was significantly correlated with increased expression of Snail1 (p = 0.03) and Snail2 (p = 0.019) but not with Twist and Foxc2 expression.

In the present series we did not observe the “cadherins switch”. N-cadherin expression not only did not increase in malignant tumors but, on the contrary, it decreased in parallel to E-cadherin expression.

### Survival analysis

**Figure 4:**
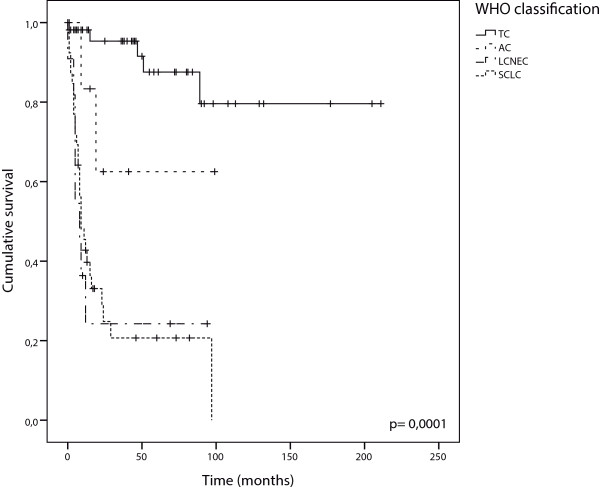
**Cumulative Kaplan-Meier survival curves stratified according to WHO classification.**

The mean follow-up time for this cohort was 90 months, with a range of 12-242 months and the five year survival rate was 53.8%. The five year cumulative survival rate for patients stratified by NET morphologic subtypes was significantly higher in TC and AC [87.6% and 62.5%, respectively] than in the LCNECs and SCLC [24.2% and 20.7%, respectively] (Figure 4).

**Figure 5:**
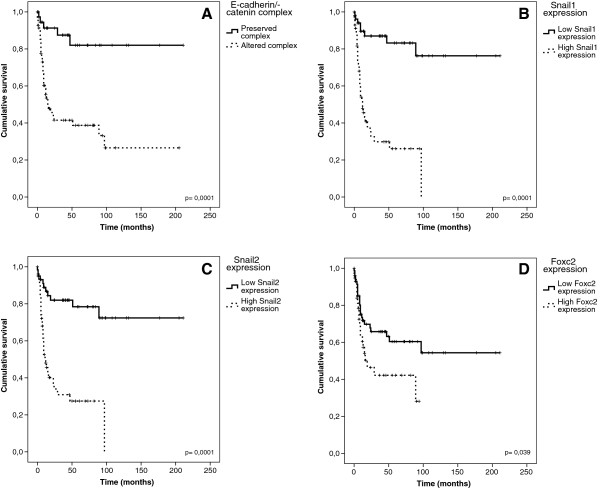
**Cumulative Kaplan–Meier survival curves stratified according to: A: E-cadherin/β-catenin complex, B, Snail1 protein expression levels, C: Snail2 protein expression levels and D: Foxc2 protein expression levels.**

A negative impact on patient survival was found for the following clinicopathological variables: gender (male), age (>56 years), node involvement, tobacco consumption and high mitotic index. Among these, tobacco consumption presented the highest Hazard Ratio (11-fold risk of death of disease). Regarding molecular variables, those with a negative impact on survival were: altered E-cadherin/β-catenin complex (Figure 5A), high Snail1 (Figure 5B), Snail2 (Figure 5C) and Foxc2 levels, (Figure 5D). Among them, Snail1 presented the highest Hazard Ratio (7-fold risk of death of disease) (Table 2).

**Table 2:** **Results of univariate and multivariate Cox survival analysis**

***Univariate Cox analysis***	Sig.	HR	95% CI	
Gender (male)	0.001	4.43	1.86	10.58
Age (>56 years)	0.005	2.61	1.03	1.08
Lymph Node (affected)	0.000	6.63	3.38	12.99
Mitotic index (high)	0.000	4.15	2.51	6.88
Tobacco consumption	0.000	11.18	3.44	36.31
Diagnosis	0.000	2.23	1.69	2.94
Necrosis (present)	0.000	8.95	3.6	22.24
Snail1 (high expression)	0.000	6.96	3.17	15.28
Snail2 (high expression)	0.000	5.2	2.57	10.54
Foxc2 (high expression)	0.039	1.85	1.01	3.38
E-cadherin (low expression)	0.001	3.04	1.54	6.03
β-catenin (low expression)	0.052	1.88	1	3.56
E-cadherin/β-catenin complex integrity (lost)	0.000	5.78	2.27	14.72
***Multivariate Cox analysis***				
Age (>56 years)	0.029	1.029	1	1.06
Diagnosis	0.006	1.634	1.15	2.33
Tobacco consumption	0.024	4.839	1.23	18.99

In multivariate survival analysis (Cox), we included the following variables, which were significant in univariate analysis: age, gender, tobacco consumption, diagnosis, lymph node involvement, mitotic index and all molecular variables regarding protein expression. Age, diagnosis and tobacco consumption showed an independent prognostic value (Table 2).

### Differences in survival between TCs

**Figure 6:**
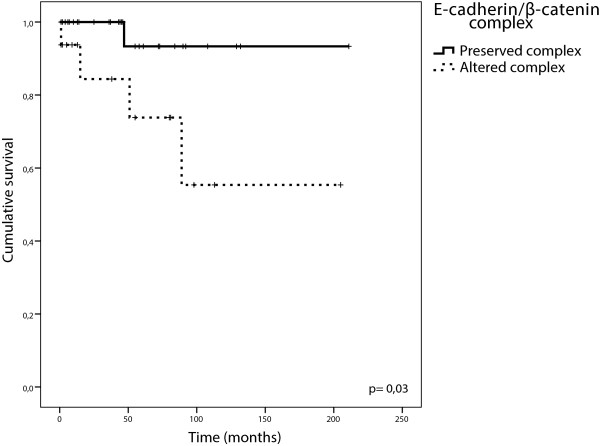
**Cumulative Kaplan–Meier survival curves stratified of patients diagnosed with TC, according to E-cadherin/β-catenin complex.**

Typical carcinoid (TC) tumors have better prognosis than other pulmonary NETs. However, some of these patients follow an unfavourable clinical course, unpredictable from the available clinicopathological features, including lymph node status. In our series, only 1/5 patients with an unfavourable course had affected nodes. With the aim of finding some molecular factors that could help in the identification of this subgroup, we performed a survival analysis on the TC series. The mean follow-up time for this group was 106 months (range 14-242 months) and 92.4% had tumour free lymph nodes. There was a difference in ten year survival rates when these patients were stratified by E-cadherin/β-catenin complex integrity (94% complex preserved vs 56% complex altered p = 0.03) (Figure 6). This result could provide tools that assist the clinician to identify patients with TC of poor prognosis.

## Discussion

Pulmonary NETs are considered a rare pathology. Despite our knowledge about their biology and genetics has increased in the past years, the mechanisms or pathways involved in the progression and metastasis still remain unclear. One of the most studied mechanisms of tumor cell spread is the Epithelial Mesenchymal Transition (EMT), which has been observed in breast [23], ovarian, colon [24] and esophageal [25] cancer models. The first step in EMT process is the loss of E-cadherin/β-catenin complex. Accordingly, in our study we observed that the E-cadherin/β-catenin complex was localised in the cellular cytoplasmatic membranes with a linear pattern and a high level expression of both molecules in the normal tissues used as controls. Such expected observations are consistent with this complex’s function in mediating intercellular adherens junctions, maintaining the integrity of the epithelium [26]. However, this pattern was lost in tumors, where a cytoplasmic localization of these molecules was observed. Moreover, reduced membrane expression levels of E-cadherin and β-catenin correlated with tumors of higher malignancy, of greater size, higher mitotic index and with necrosis. Importantly, loss of complex integrity was the molecular factor conferring a higher risk of lymph node involvement (5,6-fold) in an independent fashion. These results, in concordance with other studies [27, 28], support the notion of *CDH1* gene functioning as an invasion suppressor gene in this type of tumor.

Although it was not our initial focus, we considered the β-catenin localization when absent from the cell membrane. It is known that β-catenin has two important roles in tumorigenesis: through its interaction with E-cadherin at the cell surface it allows the formation of intercellular adherens junctions, mediating contact inhibition and suppressing tumor invasion. However, β-catenin is also a key mediator in the Wnt signalling pathway which regulates cell proliferation and differentiation [29, 30].

Activation of the Wnt pathway leads to a rise in β-catenin levels in the cytoplasm, and its accumulation in the nucleus. There, it activates the TCF/LEF transcription factors, which act on a number of Wnt target genes, including c-Myc, tcf1 and cyclinD1. The role of the Wnt/β-catenin pathway has been established in a number of tumor types, particularly in the development of colorectal carcinoma and other carcinomas. However, there is limited knowledge of its role in mesenchymal tumors. Interestingly, in our pulmonary NET series, 31 cases displayed cytoplasmic β-catenin signal and in 8 additional cases a nuclear signal was detected. Taken together, most of these were SCLC samples (23/39) (p < 0.0001), suggesting a possible contribution of cytoplasmic/nuclear β-catenin to pulmonary NET aggresiveness. This observation merits further studies to elucidate the potential involvement of the Wnt signaling pathway to pulmonary NET development.

With the purpose of deepening into the knowledge of the molecular events associated with the of E-cadherin/β-catenin complex loss of integrity in pulmonary NETs, we evaluated the expression of transcription factors, Snail1, Snail2, Twist and Foxc2, that have been shown to be involved in EMT. Healthy respiratory epithelium showed negative immunostaining for these factors. However, pulmonary NETs showed Snail1 and Snail2 expression, with an inverse correlation with E-cadherin expression consistent with their role as transcriptional repressors of E-cadherin [14, 31]. Irrespective of the other factors’ expression levels, high Snail1 protein nuclear expression conferred a higher risk of losing E-cadherin/β-catenin complex integrity in the membrane and a higher risk of finding N-cadherin in the cytoplasm. Likewise, Twist expression correlated with N-cadherin expression.

The observation of an association between Vimentin and Snail1 or Snail2 high expression confirms the results described by Kokkinos et al. as a key event in the process of EMT *in vitro* and *in vivo* [32].

Importantly, Snail1, Snail2, Foxc2, E-cadherin, N-cadherin, β-catenin expression and complex integrity all showed an impact on disease prognosis, with high Snail expression and loss of complex integrity being the factors with strongest effect in the five-year survival rates (7-fold and 6-fold higher risk of death of disease, respectively). Among clinicopathological variables, diagnosis, age and tobacco consumption were the factors with independent prognostic value. These results are in concordance with those by Travis et al [33].

The *“cadherin switch”* described in embryonic development, carcinogenesis and metastasis is characterized by a switch in classical cadherins yielding high levels of N-cadherin (*CDH2* gene) regardless of E-cadherin levels (*CDH1* gene) [34]. In our series, N-cadherin expression was detected as a faint signal in some epithelial healthy cells as a reminiscence of its expression during embryonic development [35]. In tumors, N-cadherin protein expression did not increase with malignancy, on the contrary, it was expressed at high levels in TCs and decreased in parallel with E-cadherin expression. Elevated protein levels of both cadherins were observed more frequently in tumors of low malignant potential (TCs) and favourable clinical features. These results, along with others from other authors [36, 37], might suggest that N-cadherin does not contribute to the aggressive behaviour of pulmonary NETs, but might serve as a marker of neuroendocrine differentiation. Thus, differentiated tumors might maintain N-cadherin expression that would be lost in dedifferentiated aggressive tumours. In line with our results, N-cadherin has been shown to be expressed in other tumor types from neural crest/neuroendocrine cells origin such as astrocytomas [38], Merkel cell carcinoma [39] and pheochromocytomas and adrenal tumors [40] as well as in our recent study in gastroenteropancreatic neuroendocrine tumors [41]. According to these results, the presence of N-cadherin in pulmonary neuroendocrine tumors may be due to neuroendocrine differentiation of the precursor cell and does not appear to convey aggressive behavior as demonstrated in other non-neuroendocrine malignancies. Our results support the notion proposed by Zynger et al [37] that N-cadherin would be a reliable immunohistochemical marker for these tumors.

In our series, determination of altered complex integrity turned out to be of help in AC vs TC discrimination. The loss of the linear membrane pattern is a clear indicative of complex alteration. Assessing loss of N-cadherin as well could reinforce that discrimination. However, further studies aimed at confirming this utility are needed in order to propose these determinations to be included in the clinicopathological practice.

Our findings in high grade lung neuroendocrine tumors, SCLC and LCNEC, are in favour to keep them as separate entities. In spite of their clinical and molecular similarities, their expression of adhesion molecules, low E-cadherin and β-catenin cytoplasmic expression levels, as well as high Snail1, Snail2 and Twist expression may have a value in the differential diagnosis of SCLC and LCNEC.

The Carcinoid subtype is a quite separate entity from the high-grade NETs and constitutes 1-2% of lung tumors [42, 43]. However, some TCs cases with favourable pathologic features follow an unpredictable unfavourable clinical course. To elucidate whether some of the studied markers could be of help in the identification of this subgroup of patients, we assessed the impact of clinicopathological parameters and the molecular alterations in survival rates of TCs group. We noted that when the E-cadherin/β-catenin complex was altered, the ten year survival rate was 56%, versus 94% of those patients whose tumors retained complex integrity. Loss of E-cadherin/β-catenin integrity has been described as one of the first events preceding tumor invasion. In line with this, its evaluation has turned to be of help in distinguishing TC cases that have already initiated the EMT program, thus conferring more aggressiveness and a worse prognosis. These results highlight the potential value of the immunohistochemical determination of E-cadherin/β-catenin complex status to identify patients with TCs of poor prognosis. From our perspective, this is one outstanding finding of this report.

## Conclusions

In conclusion, our study suggests that loss of E-cadherin/β-catenin complex integrity is an early event in pulmonary NETs progression and constitutes an independent predictor of lymph node involvement and reduced survival in pulmonary NETs, along with Snail1 and Twist expression, mitotic index and tobacco consumption. Immunohistochemical determination of the complex integrity might be a useful molecular tool of potential clinical application in the management of pulmonary NETs, helping in the differential diagnosis of AC vs TCs and providing information about prognosis, especially among patients with TCs, identifying the subgroup with worse prognosis despite favourable pathological features.

## Electronic supplementary material

Additional file 1: **A-D, Ki67 immunostaining (400X, scale bar de 20 μm) and E-H, H&E stains (200X, scale bar 50 μm) for each NET type are shown (A, E, TC; B, F, AC; C, G, LCNEC; D, H, SCLC).** (JPEG 1 MB)

Additional file 2: **The interobservers and intraobserver reproducibility of IHC staining scoring.** (PDF 56 KB)

Additional file 3: **Nuclear β-catenin immunostaining in LCNEC (A; 400X, scale bar de 20 μm) and SCLC (B; 200X, scale bar 50 μm).** (TIFF 4 MB)
