# Supplementary figures and images for: Prognostic and diagnostic value of epithelial to mesenchymal transition markers in pulmonary neuroendocrine tumors
Source: BMC Cancer. 2014 Nov 20;14:855. doi: 10.1186/1471-2407-14-855 (PMC4256901; doi:10.1186/1471-2407-14-855)

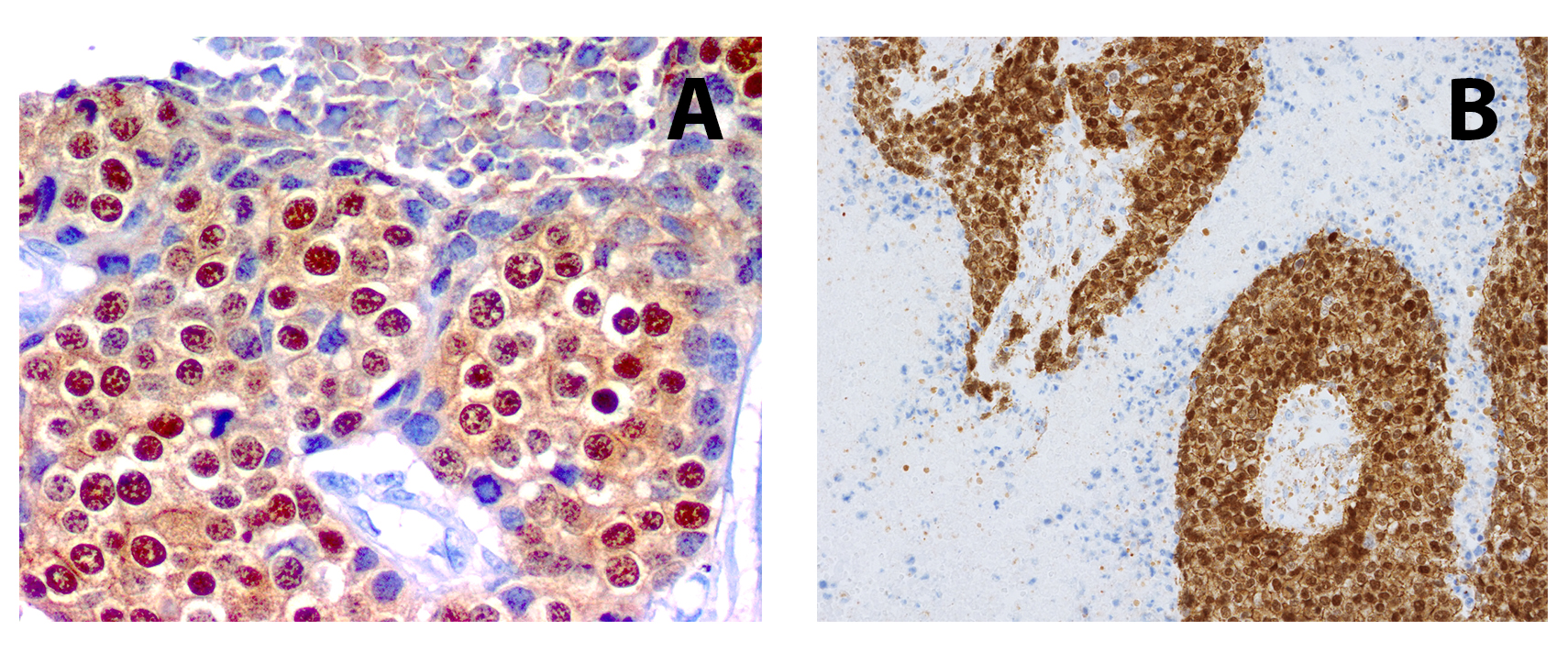

Supplement: Supplementary file 1 — Additional file 1: A-D, Ki67 immunostaining (400X, scale bar de 20 μm) and E-H, H&E stains (200X, scale bar 50 μm) for each NET type are shown (A, E, TC; B, F, AC; C, G, LCNEC; D, H, SCLC). (JPEG 1 MB) [file 12885_2013_5034_MOESM1_ESM.jpeg]

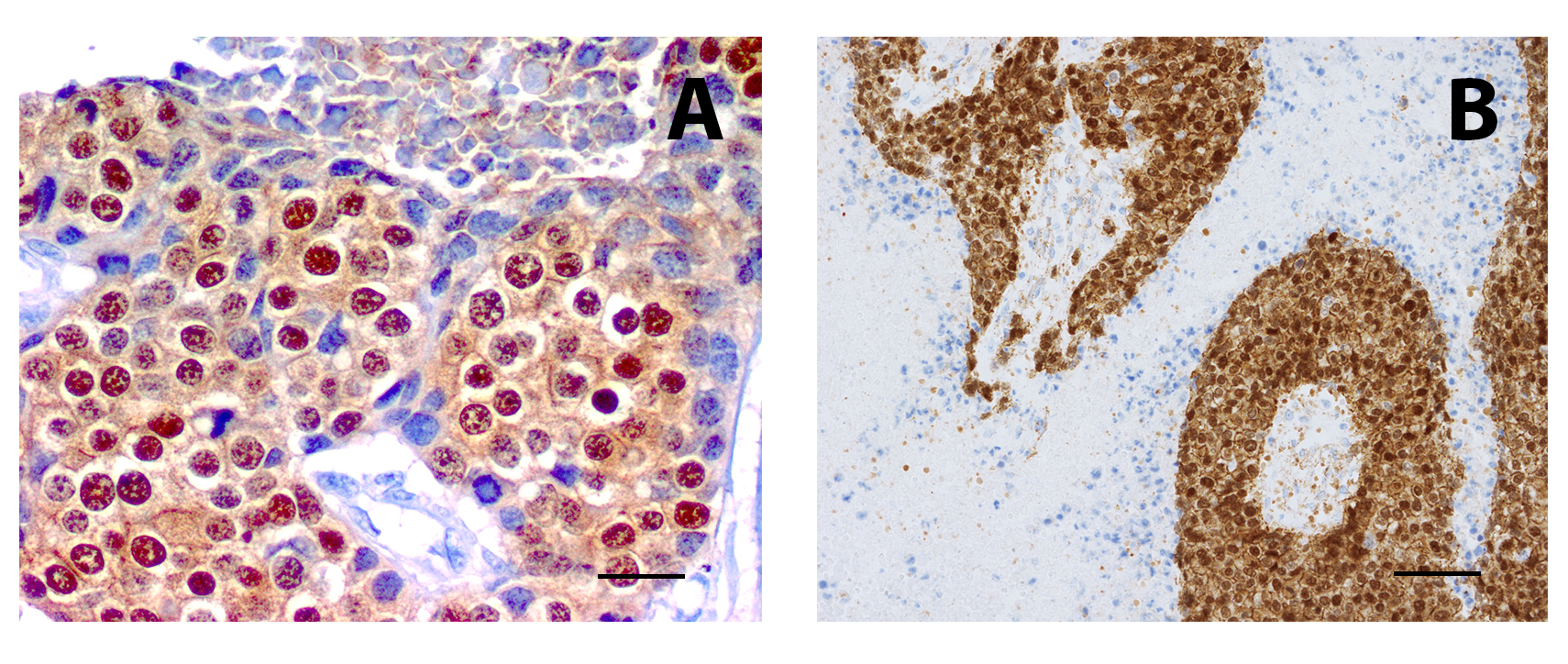

Supplement: Supplementary file 3 — Additional file 3: Nuclear β-catenin immunostaining in LCNEC (A; 400X, scale bar de 20 μm) and SCLC (B; 200X, scale bar 50 μm). (TIFF 4 MB) [file 12885_2013_5034_MOESM3_ESM.tiff]
