# Supplementary material for: Prognostic and diagnostic value of epithelial to mesenchymal transition markers in pulmonary neuroendocrine tumors
Source: BMC Cancer. 2014 Nov 20;14:855. doi: 10.1186/1471-2407-14-855 (PMC4256901; doi:10.1186/1471-2407-14-855)
Supplement: Supplementary file 2 — Additional file 2: The interobservers and intraobserver reproducibility of IHC staining scoring. (PDF 56 KB) [file 12885_2013_5034_MOESM2_ESM.pdf]

The interobservers reproducibility of scoring IHC staining has been determined by Kappa statistics for all markers assessed by 2 independent observers. The kappa statistic, defined as the difference between the observed agreements and the agreements expected by chance expressed as a fraction of the maximum possible difference[1] was applied to processed scoring data: ‘low’ vs. ‘high’ protein expression levels or ‘positive’ vs. negative or ‘membrane vs. cytoplasmic vs. nuclear immunolocalization’.

A Kappa score <0.20 is regarded as poor, a Kappa score between 0.2 and 0.40 is regarded as fair, a Kappa score between 0.4 and 0.6 is regarded as moderate, a Kappa score between 0.6 and 0.8 is regarded as substantial between observers and a Kappa score > 0.8 represents an excellent agreement.

1. Landis JR, Koch GG: **The measurement of observer agreement for categorical data**. *Biometrics* 1977, **33**(1):159-174.

Supplementary table 1: Inter-observers reproducibility for molecular variables measured by Kappa statistic

Variable	Kappa	Standard error	Sig.	CI 95%
E-cadherin protein expression	0,894	0,039	0,000	0,819-0,970
E-cadherin localization	0,816	0,052	0,000	0,714-0,918
β-catenin protein expression	0,746	0,057	0,000	0,634-0,859
β -catenin localization	0,788	0,060	0,000	0,672-0,905
N-cadherin protein expression	0,881	0,041	0,000	0,800-0,961
N-cadherin localization	0,822	0,056	0,000	0,711-0,932
Snail1 protein expression	0,894	0,039	0,000	0,818-0,970
Snail2 protein expression	0,878	0,042	0,000	0,796-0,960
Twist1 protein expression	0,880	0,041	0,000	0,799-0,961
FoxC2 protein expresion	0,832	0,034	0,000	0,734-0,866
Vimentin protein expression	0,826	0,120	0,000	0,590-1,062

Supplementary table 2: Intra-observers reproducibility for molecular variables measured by Kappa statistic

	OBSERVER 1				OBSERVER 2			
**Variable**	Kappa	Standard error	Sig.	CI 95%	Kappa	Standard error	Sig.	CI 95%
E-cadherin protein expression	0.940	0.030	0.000	0.875-0.985	0.954	0.027	0.000	0.893-0.998
E-cadherin localization	0.901	0.039	0.000	0.818-0.967	0.965	0.025	0.000	0.910-0.999
β-catenin protein expression	0.925	0.032	0.000	0.863-0.985	0.955	0.026	0.000	0.895-0.990
β -catenin localization	0.946	0.030	0.000	0.877-0.999	0.914	0.040	0.000	0.823-0.980
N-cadherin protein expression	0.955	0.025	0.000	0.896-0.998	0.955	0.027	0.000	0.895-0.999
N-cadherin localization	0.919	0.040	0.000	0.830-0.980	0.914	0.040	0.000	0,823-0,980
Snail1 protein expression	0.955	0.026	0.000	0.895-0.998	0.938	0.030	0.000	0.874-0.985
Snail2 protein expression	0.863	0.045	0.000	0,765-0.940	0.924	0.034	0.000	0.849-0.984
Twist1 protein expression	0.940	0.029	0.000	0,879-0.985	0.955	0.033	0.000	0.860-0.984
FoxC2 protein expresion	0.983	0.026	0.000	0.942-0.999	0.932	0.033	0.000	0.860-0.984
Vimentin protein expression	1	0.000	0.000	1,000-1,000	1	0.000	0.000	1,000-1,000
